# Impact of Proestrus on Gene Expression in the Medial Preoptic Area of Mice

**DOI:** 10.3389/fncel.2017.00183

**Published:** 2017-07-04

**Authors:** Csaba Vastagh, Zsolt Liposits

**Affiliations:** ^1^Laboratory of Endocrine Neurobiology, Institute of Experimental Medicine, Hungarian Academy of SciencesBudapest, Hungary; ^2^Department of Neuroscience, Faculty of Information Technology and Bionics, Pázmány Péter Catholic UniversityBudapest, Hungary

**Keywords:** AVPV, MPOA, mouse, proestrus, metestrus, gene expression, PCR, immunocytochemistry

## Abstract

The antero-ventral periventricular zone (AVPV) and medial preoptic area (MPOA) have been recognized as gonadal hormone receptive regions of the rodent brain that—via wiring to gonadotropin-releasing hormone (GnRH) neurons—contribute to orchestration of the preovulatory GnRH surge. We hypothesized that neural genes regulating the induction of GnRH surge show altered expression in proestrus. Therefore, we compared the expression of 48 genes obtained from intact proestrous and metestrous mice, respectively, by quantitative real-time PCR (qPCR) method. Differential expression of 24 genes reached significance (*p* < 0.05). Genes upregulated in proestrus encoded neuropeptides (kisspeptin (KP), galanin (GAL), neurotensin (NT), cholecystokinin (CCK)), hormone receptors (growth hormone secretagogue receptor, μ-opioid receptor), gonadal steroid receptors (estrogen receptor alpha (ERα), progesterone receptor (PR), androgen receptor (AR)), solute carrier family proteins (vesicular glutamate transporter 2, vesicular monoamine transporter 2), proteins of transmitter synthesis (tyrosine hydroxylase (TH)) and transmitter receptor subunit (AMPA4), and other proteins (uncoupling protein 2, nuclear receptor related 1 protein). Proestrus evoked a marked downregulation of genes coding for adenosine A2a receptor, vesicular gamma-aminobutyric acid (GABA) transporter, 4-aminobutyrate aminotransferase, tachykinin precursor 1, NT receptor 3, arginine vasopressin receptor 1A, cannabinoid receptor 1, ephrin receptor A3 and aldehyde dehydrogenase 1 family, member L1. Immunocytochemistry was used to visualize the proteins encoded by *Kiss1, Gal, Cck* and *Th* genes in neuronal subsets of the AVPV/MPOA of the proestrous mice. The results indicate that gene expression of the AVPV/MPOA is significantly modified at late proestrus including genes that code for neuropeptides, gonadal steroid hormone receptors and synaptic vesicle transporters. These events support cellular and neuronal network requirements of the positive estradiol feedback action and contribute to preparation of the GnRH neuron system for the pre-ovulatory surge release.

## Introduction

The antero-ventral periventricular zone (AVPV) and medial preoptic area (MPOA) referred as AVPV/MPOA, are conspicuous constituents of the rostro-ventral hypothalamus of the rodent brain. This region has a pivotal role in central regulation of reproduction (Barr and Barraclough, 1978; Petersen et al., 1989) and control of paternal behavior (Lee and Brown, 2002; Paredes, 2003; Hull and Dominguez, 2007; Tsuneoka et al., 2013; Akther et al., 2014) and social reward (McHenry et al., 2017). It shows the signs of sexual dimorphism by displaying a considerably larger volume and rostro-caudal extent in females than males (Davis et al., 1996). The area has been identified as a main target of gonadal hormone actions. Accordingly, functional estrogen receptor alpha (ERα) and beta (ERβ; Ehret and Buckenmaier, 1994; Merchenthaler et al., 2004; Bodo et al., 2006; Sano et al., 2013; Zuloaga et al., 2014; Nakata et al., 2016), progesterone receptor (PR; Simerly et al., 1996; Kudwa et al., 2004) and androgen receptor (AR; Simerly et al., 1990) have been described in the region. Its significance in reproduction was reinforced by the pioneer finding that medial preoptic micro-implants of the non-steroidal antiestrogen, Keoxifene blocked the afternoon luteinizing hormone (LH) surge (Petersen et al., 1989). The chemical phenotyping of the area revealed the production of a wide scale of neuropeptides and the synthesis of certain neurotransmitters. Subsets of neurons produce kisspeptin (KP; Smith et al., 2005; Clarkson and Herbison, 2006; Ducret et al., 2010; Overgaard et al., 2013; Kumar et al., 2015; Skrapits et al., 2015; Yip et al., 2015; Yeo et al., 2016), galanin (GAL; Porteous et al., 2011; Wu et al., 2014), cholecystokinin (CCK; Simerly and Swanson, 1987), substance-P (SP; Simerly and Swanson, 1987; Okamura et al., 1994), met-enkephalin (ENK; Simerly and Swanson, 1987; Porteous et al., 2011) and neurotensin (NT; Simerly and Swanson, 1987; Dungan Lemko et al., 2010) representing the peptidergic profile. Synthesis of gamma-aminobutyric acid (GABA; Cravo et al., 2011; Liu and Herbison, 2011; Liu et al., 2011; Cheong et al., 2015), glutamate (Cravo et al., 2011; Liu et al., 2011; Cheong et al., 2015) and dopamine (DA; Simerly and Swanson, 1987; Simerly et al., 1997; Clarkson and Herbison, 2011) in the area proves its capability of networking via neurotransmitter signaling. Neurons with dual phenotype have also been identified in the region (Ottem et al., 2004; Clarkson and Herbison, 2011; Skrapits et al., 2015). Tract tracing studies explored the neuronal projections of the AVPV/MPOA to various loci of the central nervous system indicating its involvement in the control of various neuronal networks (Simerly and Swanson, 1988; Gu and Simerly, 1997; Simerly, 1998).

Regarding its role in the central regulation of reproduction, elucidation of wiring of AVPV/MPOA neurons with gonadotropin-releasing hormone (GnRH) neurons, identification of putative regulatory neurotransmitters and neuropeptides in the established connections and clarification of their regulatory influence upon GnRH network-related functions are indispensable. In mice, the expression of the trans-neuronal genetic tracer, barely lectin (BL) in GnRH neurons allowed the identification of their afferent neuronal systems including projections from the AVPV and MPOA (Boehm et al., 2005). The use of genetic trans-synaptic tracing strategy has also proved that KP afferents of AVPV origin innervate GnRH neurons, the connection is estrogen sensitive and utilizes DA as co-modulator (Kumar et al., 2015). Subsets of KP neurons in the rostral periventricular regions of the third ventricle (RP3V) co-synthesize GAL and ENK (Porteous et al., 2011). CCK and NT do not seem to act as co-modulators in KP neurons (Porteous et al., 2011). Signaling to GnRH neurons by KP (Han et al., 2005; Clarkson et al., 2008; Kalló et al., 2012), GAL (Kalló et al., 2012), CCK (Giacobini and Wray, 2007), NT (Dungan Lemko et al., 2010) and ENK (Porteous et al., 2011) has also been revealed in rodents. Regarding the neurotransmitter signaling, glutamate- and GABA-ergic inputs to GnRH cells arising from the AVPV/MPOA have been shown (Cravo et al., 2011) and their impact on GnRH neuron physiology confirmed by electrophysiology (Han et al., 2002; Iremonger et al., 2010; Moenter, 2010; Liu and Herbison, 2011). Likewise, the modulation of GnRH neuron by DA (Liu and Herbison, 2013) has also been established.

The participation of estradiol (E2)-receptive neuron populations of the AVPV/MPOA in mediation and execution of the positive E2 feedback effect has been confirmed (Wintermantel et al., 2006; Christian et al., 2008; Moenter et al., 2009; Dubois et al., 2015). Evidences indicate that circulating E2 activates ER-alpha expressing KP neurons in the MPOA in proestrus (Smith et al., 2006b; Adachi et al., 2007; Clarkson et al., 2008; Clarkson and Herbison, 2009) by modulating ionic currents of these neurons in an estrous cycle dependent manner (Piet et al., 2013).

These observations undoubtedly indicate that the AVPV/MPOA has a substantial contribution to regulation of the GnRH system by relaying the hormonal message of the positive E2 feedback during proestrus. Accordingly, it is conceivable to assume that E2 modifies the transcriptome of the RP3V area preceding the preovulatory GnRH surge. The present study was aimed to monitor proestrus-regulated changes in the transcriptome of the AVPV/MPOA in gonadally intact, cycling female mice obtained from proestrus and metestrus stages of the ovarian cycle, respectively.

## Materials and Methods

### Ethics Statement

All experiments were performed with permissions from the Animal Welfare Committee of the Institute of Experimental Medicine Hungarian Academy of Sciences (Permission Number: A5769-01) and in accordance with legal requirements of the European Community (Directive 2010/63/EU). All animal experimentation described was conducted in accordance with accepted standards of humane animal care and all efforts were made to minimize suffering.

### Animals

Adult, gonadally intact C57BL/6J female mice (*n* = 20) were used from local colonies bred at the Medical Gene Technology Unit of the Institute of Experimental Medicine (IEM). They were housed in light (12:12 light-dark cycle, lights on at 06:00 h)—and temperature (22 ± 2°C) controlled environment, with free access to standard food and tap water. The estrous cycle was monitored daily between 9 a.m. and 10 a.m. by microscopic evaluation of vaginal cytology (Nelson et al., 1982; Byers et al., 2012; Cora et al., 2015). Proestrous and metestrous female mice with at least two consecutive, regular estrous cycles were used. To avoid the circadian effect, animals were sacrificed between 16:00 h and 18:00 h. We have recently described the biomarkers of these animals reporting that late-proestrous mice are characterized by predominantly nucleated epithelial cells in the vaginal smear, increased serum luteinizing (LH) hormone level (>5 mg/L), enlarged uterine weight (>0.15 g) and higher burst activity and firing frequency of GnRH neurons (Farkas et al., 2013; Vastagh et al., 2016) in comparison with metestrous animals. These observations are in good agreement with other reports characterizing the bio-profile of intact proestrous mice (Murr et al., 1973; Czieselsky et al., 2016; Silveira et al., 2017). The uterine wet weight correlates with serum LH level, therefore, it is considered as a reliable marker of the late proestrous mice developing the pre-ovulatory GnRH surge (Vastagh et al., 2016; Silveira et al., 2017) Accordingly, the uterine weights clearly differentiated between the proestrous and metestrous experimental groups displaying 0.155 ± 0.013 g and 0.076 ± 0.008 g values, respectively.

### Dissection of the AVPV/MPOA Blocks from Mouse Hypothalami

Proestrous (*n* = 6) and metestrous (*n* = 6) mice were deeply anesthetized, decapitated and then brains were rapidly removed and placed into a pre-chilled brain matrix. A 1 mm-thick coronal slice—extending between Bregma +0.9 mm and Bregma −0.1 mm as antero-posterior coordinates—was cut from each brain. The bilateral AVPV/MPOA blocks were dissected in a triangle-shape as shown in Figure 3. Tissue blocks were collected individually from the brains and placed immediately in RNAlater (Thermo Fisher Scientific, Waltham, MA, USA) and stored at −80°C until further use.

**Figure 1:**
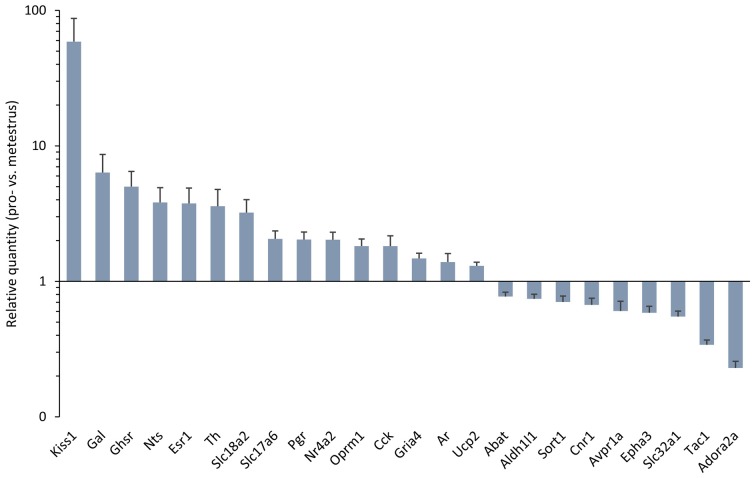
Differentially expressed genes in the antero-ventral periventricular/medial preoptic area (MPOA) of the late proestrous mouse. Relative quantity (RQ) values indicate the relative expression of genes in the pro- vs. metestrous experimental groups (*p* < 0.05, Student’s *t*-test). RQ values are visualized as a graph with log10 scale on the *y* axis.

**Figure 2:**
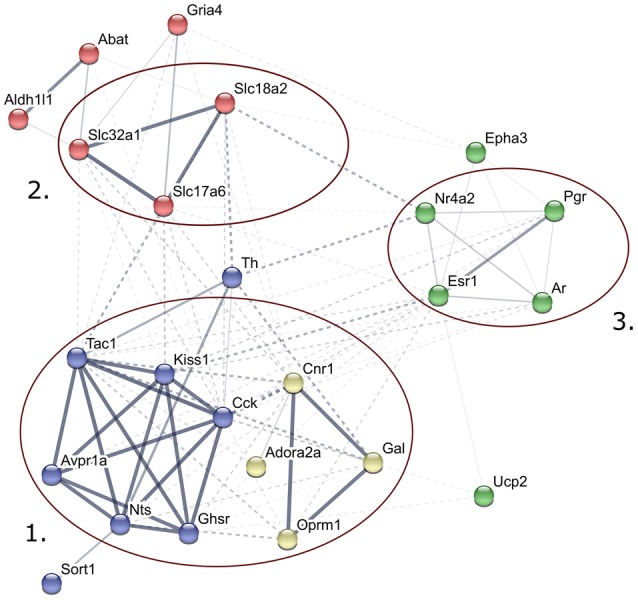
Predicted interactions among proteins encoded by genes expressed differentially in late proestrus. Network visualization of the differentially expressed proteins was achieved by STRING v10 (http://string-db.org). The clusters of neuropeptides/neuropeptide receptors coupled to G proteins (1), solute carrier proteins involved in synaptic vesicle cycle (2) gonadal steroid hormone signaling pathway (3) are prominent.

**Figure 3:**
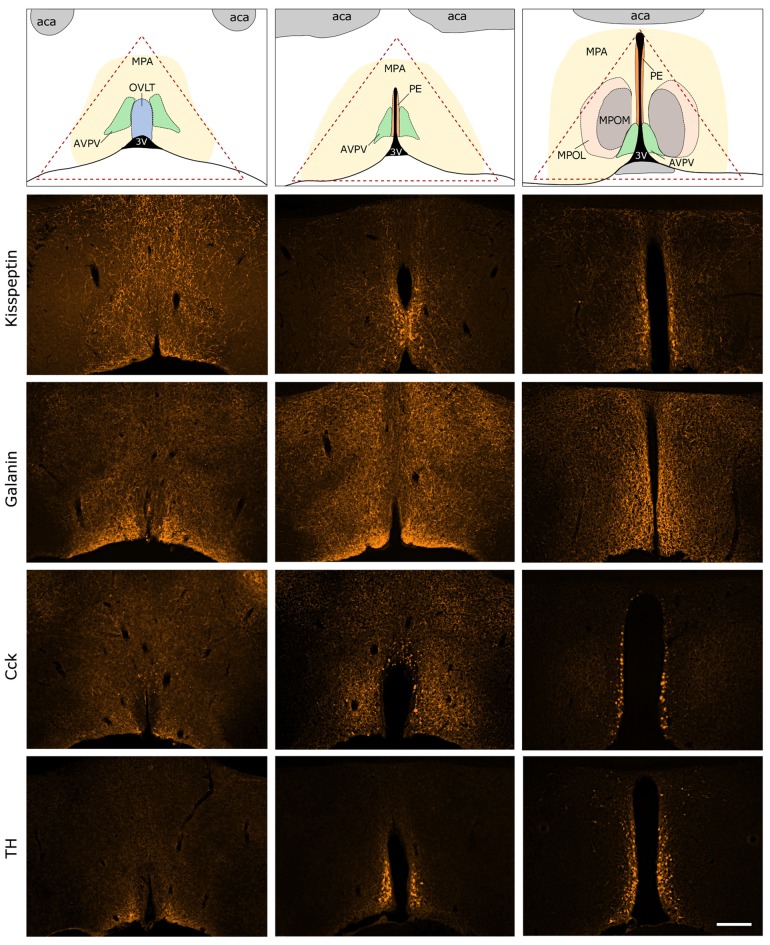
Immunocytochemical localization of kisspeptin (KP), galanin (GAL), cholecystokinin (CCK) and tyrosine-hydroxylase (TH) in the antero-ventral periventricular/MPA of the proestrous mouse. The uppermost row illustrates the MPA and its subdivisions in three representative rostro-caudal planes (from left to right). Red triangle indicates the border lines of tissue area sampled for the real-time PCR study. Immunofluorescent detection of KP, galanin, CCK and TH is provided in the three, representative frontal planes. KP, CCK and TH appear in cell bodies within the AVPV and the periventricular nucleus (PE) in addition to neuronal processes. Galanin-immunoreactivity is not detectable at the level of perikarya in proestrous mouse, despite its intense occurrence in axons. aca, anterior commissure; AVPV, antero-ventral periventricular nucleus; MPOM, medial division of the medial preoptic nucleus; MPOL, lateral division of the medial preoptic nucleus; OVLT, vascular organ of lamina terminalis; 3V, 3rd ventricle. Scale bar: 200 μm.

### RNA Isolation, Reverse Transcription and Pre-Amplification

Dissected tissue blocks containing the AVPV/MPOA were shredded in RLT buffer (Qiagen, Hilden, Germany) supplemented with 1% beta-mercaptoethanol. Total RNA was isolated from each individual AVPV tissue sample using RNeasy Micro kit (Qiagen, Hilden, Germany) according to manufacturer’s protocol. Genomic DNA was eliminated by treatment with 1 U of RNase-free DNase-I (Thermo Fisher Scientific). Total RNA was eluted with 14 μl of ribonuclease-free water. For integrity analysis, RNA was measured using RNA Pico Chip on the 2100 Bioanalyzer (Agilent, Santa Clara, CA, USA). Twenty-five nanograms column-purified RNA were reverse transcribed per samples using SuperScript VILO cDNA synthesis kit (Thermo Fisher Scientific). The resultant cDNA product was pre-amplified with TaqMan PreAmp Master Mix Kit (Thermo Fisher Scientific) according to the manufacturer’s protocol.

### Quantitative Real-Time PCR Studies

For quantitative real-time PCR (qPCR) investigations of the AVPV/MPOA samples (proestrous females *n* = 6, metestrous females *n* = 6) pre-amplified cDNA diluted in 0.1× TE buffer was used as template for qPCR. Inventoried TaqMan assays (Thermo Fisher Scientific) were used in the qPCR investigation. Each assay consisted of a FAM dye-labeled TaqMan MGB probe and two PCR primers. Thermal cycling conditions of the qPCR were as follows: 2 min at 50°C and 10 min at 95°C, followed by 40 cycles of 15 s at 95°C and 1 min at 60°C using ViiA 7 real-time PCR platform (Thermo Fisher Scientific). Using the geometric mean of cycle threshold (Ct) values of the reference genes (*Gapdh* and *Hprt*), the relative gene expression (RQ) was calculated by the 2^−ΔΔCt^ method (Livak and Schmittgen, 2001) where ΔΔCt = (Ct_target_ − Ct_reference_)_proestrus_ − (Ct_target_ − Ct_reference_)_metestrus_.

### Statistical Analysis

Delta Ct values (ΔCt; Ct_target_ − Ct_reference_) for proestrus and metestrus groups were subjected to two-tailed Student’s *t*-test to estimate ΔΔCt in comparison of gene expression. RQs were expressed as mean ± SEM, with number of animals *n* = 4 per groups. Differences were considered as significant when *p* < 0.05.

### Immunocytochemistry

Adult female mice (*n* = 8) were deeply anesthetized and perfused transcardially with 4% paraformaldehyde in PBS (pH = 7.4) in their proestrus phase between 16:00 h and 18:00 h. Brains were removed from the skulls and post-fixed for 1 h at RT, then equilibrated in 30% sucrose-PBS. Thirty micrometer thick sections were cut in the coronal plane using a freezing microtome (SM2000R, Leica Biosystems, Nussloch, Germany). The sections were washed several times in PBS, then permeabilized with 0.5% Triton-X100 and blocked in 2% heat-inactivated normal horse serum (NHS). Primary antisera were diluted in NHS and incubated with the sections for 72 h at 4°C. The following antisera were used: anti-KP (#AC024, 1:5000, from A. Caraty, INRA, Nouzilly, France), anti-galanin (# IS-42; 1:2000, Interchim Cat# AP101500, RRID: AB_2314518), anti-CCK-8 (# IS-15/8 RRID: AB_2314186, 1:1000, from P. Ciofi, National Institute of Health and Medical Research, Bordeaux, France) and anti-tyrosine hydroxylase (TH) (#TYH, 1:1000; Aves Labs Inc., Tigard, OR, USA). The characterization and specificity of the primary antibodies have been published elsewhere (Ciofi and Tramu, 1990; Porteous et al., 2011), the pre-absorption of the antibodies or their omission from the staining resulted in immunonegative sections. Cy3-conjugated secondary antibodies (Jackson Immunoresearch Europe Ltd, Suffolk, UK) were diluted in PBS containing 2% NHS (1:500) and applied on the sections for 4 h at RT. The sections were washed several times in PBS, then were mounted on glass slides and coverslipped in Mowiol (Sigma-Aldrich, St. Louis, MO, USA). Slides were investigated using a Zeiss Axioskop-2 (Carl-Zeiss Microscopy, Jena, Germany) microscope under ultraviolet light using filter set for Cy3 dye (excitation BP 545/25, emission BP 605/70), then sections were photographed with an AxioCam HRc digital camera controlled by AxioVision 4.6 software (Carl-Zeiss).

### Network Analysis of Differentially Expressed Genes

The list of differentially expressed genes was uploaded to a web resource (String 10.5^1^) for the analysis of known or predicted functional protein-protein interactions between proteins encoded by these genes. The scores of evidences were scaled from 0 (insignificant) to 1 in the categories as follows: neighborhood in the genome, gene fusions, co-occurrence across genomes, co-expression, experimental/biochemical data, association in curated databases and co-mentioning in PubMed abstracts. A combined score was calculated from the individual scores for each interaction. The interactions were visualized using the confidence view where connecting line thickness indicate the strength of data support. The non-hierarchical *k*-means clustering method was performed by the String.

## Results

### Network Visualization of Genes by STRING

The *k-means* clustering view of the predicted interactions among proteins encoded by the 48 target genes whose differential expression was studied are depicted in Supplementary Figure S1. The selection criteria of the 48 genes were based their known or supposed function in the regulation of the AVPV/MPOA and the estrous cycle. The official gene symbols and names are shown in Table 1.

**Table 1:** PCR analysis of genes regulated differentially in proestrus in the antero-ventral periventricular/medial preoptic area of the mouse.

Gene symbol	Description	TaqMan ID	RQ	± SEM	*p*-value
**Neuropeptides/receptors**					
Avpr1a	Arginine vasopressin receptor 1A	Mm00444092_m1	0.60	0.11	4.36E-02
Cartpt	CART prepropeptide	Mm04210469_m1	0.78	0.23	5.83E-01
Cck	Cholecystokinin	Mm00446170_m1	1.82	0.35	3.95E-02
Gal	Galanin	Mm00439056_m1	6.36	2.29	5.65E-03
Galr1	Galanin receptor 1	Mm00433515_m1	1.62	0.30	1.58E-01
Ghsr	Growth hormone secretagogue receptor	Mm00616415_m1	5.00	1.47	8.00E-03
Kiss1	KiSS-1 metastasis-suppressor	Mm03058560_m1	58.81	28.43	1.03E-02
Kiss1r	KISS1 receptor	Mm00475046_m1	0.89	0.10	3.93E-01
Nts	Neurotensin	Mm00481140_m1	3.82	1.09	8.44E-03
Oprm1	Opioid receptor, mu 1	Mm01188089_m1	1.82	0.23	2.02E-03
Sort1	Sortilin (neurotensin receptor 3)	Mm00490905_m1	0.70	0.07	1.71E-02
Sstr3	Somatostatin receptor 3	Mm00436695_s1	1.12	0.11	4.28E-01
Tac1	Tachykinin precursor 1	Mm01166996_m1	0.34	0.03	4.99E-02
**Neurotransmitter synthesis and transport**					
Abat	4-aminobutyrate aminotransferase	Mm00556951_m1	0.77	0.06	2.03E-02
Gad1	Glutamate decarboxylase 1	Mm04207432_g1	0.74	0.14	1.81E-01
Gad2	Glutamic acid decarboxylase 2	Mm00484623_m1	0.93	0.09	7.42E-01
Slc17a6	solute carrier family 17 (Vesicular Glutamate Transporter), member 1	Mm00499876_m1	2.06	0.30	1.83E-03
Slc18a2	Solute carrier family 18 (vesicular monoamine transporter), member 2	Mm00553058_m1	3.22	0.791	1.57E-02
Slc32a1	Solute carrier family 32 (GABA vesicular transporter), member 1	Mm00494138_m1	0.55	0.054	4.37E-02
Th	Tyrosine hydroxylase	Mm00447557_m1	3.58	1.19	1.43E-02
**GABA/glutamate signaling**					
Cnr1	Cannabinoid receptor 1 (brain)	Mm01212171_s1	0.67	0.08	2.31E-02
Gabbr1	Gamma-aminobutyric acid (GABA) B receptor, 1	Mm00444578_m1	1.00	0.10	9.09E-01
Gabra3	Gamma-aminobutyric acid (GABA) A receptor, subunit alpha 3	Mm01294271_m1	1.20	0.17	3.96E-01
Gabrb3	Gamma-aminobutyric acid (GABA) A receptor, subunit beta 3	Mm00433473_m1	1.12	0.14	5.28E-01
Gria1	Glutamate receptor, ionotropic, AMPA1 (alpha 1)	Mm00433753_m1	1.18	0.14	4.11E-01
Gria2	Glutamate receptor, ionotropic, AMPA2 (alpha 2)	Mm00442822_m1	0.91	0.08	8.04E-01
Gria4	Glutamate receptor, ionotropic, AMPA 4	Mm00444754_m1	1.48	0.14	1.23E-02
Grin1	Glutamate receptor, ionotropic, NMDA1 (zeta 1)	Mm00433790_m1	1.24	0.09	1.29E-01
**Gonadal steriod receptor signaling**					
Ar	Androgen receptor	Mm00442688_m1	1.39	0.21	3.02E-02
Esr1	Estrogen receptor 1 (alpha)	Mm00433149_m1	3.76	1.12	1.24E-02
Esr2	Estrogen receptor 2 (beta)	Mm00599821_m1	1.34	0.30	3.79E-01
Gper1	G protein-coupled estrogen receptor 1	Mm01194815_m1	1.11	0.15	5.22E-01
Nr4a2	Nuclear receptor subfamily 4, group A, member 2	Mm00443060_m1	2.03	0.28	4.93E-03
Pgr	Progesterone receptor	Mm00435628_m1	2.03	0.28	1.03E-03
Pgrmc1	Progesterone receptor membrane component 1	Mm00443985_m1	4.61	2.85	9.39E-02
**Others**					
Adora2a	Adenosine A2a receptor	Mm00802075_m1	0.23	0.03	2.70E-02
Amhr2	Anti-Mullerian hormone type 2 receptor	Mm00513847_m1	0.35	0.14	4.12E-01
Cxcr4	Chemokine (C-X-C motif) receptor 4	Mm01292123_m1	1.48	0.37	2.58E-01
Epha3	EPH receptor A3	Mm00580743_m1	0.59	0.07	4.16E-03
Gfap	Glial fibrillary acidic protein	Mm01253033_m1	0.71	0.09	3.64E-01
Kcnc1	Potassium voltage gated channel, Shaw-related subfamily, member 1	Mm00657708_m1	0.67	0.16	1.72E-01
Lepr	Leptin receptor	Mm00440181_m1	1.00	0.11	8.16E-01
Nrxn3	Neurexin III	Mm00553213_m1	1.37	0.26	3.15E-01
Rab14	RAB14, member RAS oncogene family	Mm00499577_m1	0.34	0.14	8.78E-01
Slc1a4	Solute carrier family 1 (glutamate/neutral amino acid transporter), member 4	Mm00444532_m1	1.17	0.14	3.40E-01
Slc25a4	Solute carrier family 25 (mitochondrial carrier, adenine nucleotide translocator), member 4	Mm01207393_m1	0.79	0.09	7.99E-02
Ucp2	Uncoupling protein 2 (mitochondrial, proton carrier)	Mm00627599_m1	1.30	0.08	1.69E-02
Aldh1l1	Aldehyde dehydrogenase 1 family, member L1	Mm03048957_m1	0.74	0.06	4.79E-02

*Forty-eight target genes were selected and grouped in neuropeptides/receptors, neurotransmitter synthesis and transport, GABA/glutamate signaling, gonadal steroid receptor signaling and others categories. Relative quantity (RQ), standard error of the mean (SEM) and p-value are given for each studied gene. Expression of 24 genes reached the level of significance (*p* < 0.05; Student’s *t*-test)*.

### Differential Expression of Genes in the AVPV/MPOA of Proestrous vs. Metestrous Mice

#### Neuropeptides and Peptide Receptors

Gene expression of neuropeptides and peptide receptors changed significantly (*p* < 0.05, Student’s *t*-test) at late proestrus compared to metestrus mice in the same period of the day. In proestrus, the relative quantity (rq; proestrus vs. metestrus) of genes increased as follows: kiss-1 metastasis-suppressor (*Kiss1*; rq 58.81 ± 28.43), galanin (*Gal*; rq 6.36 ± 2.29) growth hormone secretagogue receptor (*Ghsr*; rq 5.00 ± 1.47), neurotensin (*Nts*; rq 3.82 ± 1.09), cholecystokinin (*Cck*; rq 1.82 ± 0.35), opioid receptor mu 1 (*Oprm1*; rq 1.82 ± 0.23). Expression of arginine vasopressin receptor 1A (*Avpr1a*; rq 0.60 ± 0.11), tachykinin precursor 1 (*Tac1*; rq 0.34 ± 0.03) and sortilin/NT receptor 3 (*Sort1*; rq 0.70 ± 0.07) decreased in proestrus (Table 1 and Figure 1). No significant change has been detected in the expression of the Kiss1 receptor (*Kiss1r*), somatostatin receptor 3 (*Sstr3*), CART prepropeptide (*Cartpt*) and galanin receptor 1 (*Galr1*; Table 1).

### Neurotransmitter Synthesis and Transport; GABA and Glutamate Signaling

Expression of the L-DOPA synthetizing enzyme tyrosine hydroxylase (*Th*; rq 3.58 ± 1.19), the vesicular monoamine transporter 2 (*Slc18a2*; rq 3.22 ± 0.791), and the vesicular glutamate transporter 2 (*Slc17a6*; rq 2.06 ± 0.30) was increased in proestrus. On the contrary, decreased mRNA levels of the 4-aminobutyrate aminotransferase (*Abat*; rq 0.77 ± 0.06) and the GABA vesicular transporter 1 (*Slc32a1*; rq 0.55 ± 0.054) were measured in the proestrous mice. Expression of genes encoding GABA and glutamate receptor subunits (*Gabbr1, Gabra3, Gabrb3, Gria1, Gria2, Grin1*) remained unchanged across the comparison of the two groups, apart from the ionotropic glutamate receptor AMPA4 (*Gria4*) that showed an increased mRNA level (rq 1.48 ± 0.14) in proestrus (Table 1 and Figure 1).

### Gonadal Steroid Receptor Signaling

The estrogen receptor alpha (*Esr1*, rq 3.76 ± 1.12), the progesterone receptor (*Pgr*; rq 2.03 ± 0.28), the transcriptionally-inducible nuclear receptor (*Nr4a2*, rq 2.03 ± 0.28) and the androgen receptor (*Ar*; rq 1.39 ± 0.21) were all upregulated in proestrus (Table 1 and Figure 1). The expression level of the estrogen receptor beta (*Esr2*), the G protein-coupled estrogen receptor (*Gper1*) and the progesterone receptor membrane component 1 (*Pgrmc1*) did not change significantly (Table 1).

### Others

Expression of the uncoupling protein 2 (*Ucp2*, rq 1.3 ± 0.08) gene was increased, whereas the adenosine A2a receptor (*Adora2a*; rq 0.23 ± 0.03), the ephrin type-a receptor 3 (*Epha3*; rq 0.59 ± 0.07) and the cannabinoid receptor 1 (*Cnr1*; rq 0.67 ± 0.08) showed significant downregulated mRNA levels in proestrus (Table 1 and Figure 1). The change was not significant in the mRNA levels of the following targets: *Amhr2, Cxcr4, Gfap, Kcnc1, Lepr, Nrxn3, Rab14, Slc1a4* and *Slc25a4* (Table 1).

### Putative Interactions of Proteins Encoded by the Differentially Expressed Genes

STRING v10 database and web resource (Szklarczyk et al., 2015) allowed an interactive network visualization of proteins encoded by the 24 genes regulated differentially in proestrus (Figure 2). The footprint of proestrus was represented in protein clusters as follows: neuropeptides and neuropeptide receptors coupled to G proteins (score >0.9 in the Reactome pathways “*G alpha (q)*” and “*G alpha (i) signaling events*”), solute carrier family members (score: >0.88 in the KEGG pathway “*synaptic vesicle cycling*”) and nuclear gonadal steroid hormone receptors (combined score 0.24–0.69; enriched in GO:0043401 termed as “*steroid hormone mediated signaling pathway*”). Networking of individual proteins and the established protein clusters was observed.

### Localization of Kisspeptin, Galanin, Cholecystokinin and Tyrosine Hydroxylase Immunoreactive Structures in the AVPV/MPOA of the Proestrous Mouse

Expression of KP, GAL, CCK and TH were visualized by fluorescent immunocytochemistry in the main representative compartments of the periventricular zone in proestrous mice (Figure 3). GAL was not detectable in any perikarya, only the axons displayed GAL immunoreactivity. KP, CCK and TH were present in cell bodies located around the 3rd ventricle and in the vicinity of the vascular organ of lamina terminalis (OVLT), as well as in fine neuronal processes scattered in the medial (MPOM) and lateral (MPOL) divisions of the medial preoptic nucleus.

## Discussion

### Altered Expression of Genes Encoding Neuropeptides in the AVPV/MPOA of Proestrous Mice

The comparison of the AVPV/MPOA in met- and proestrous mice revealed the differential expression of *Kiss1, Gal, Nts* and *Cck* genes with a marked upregulation in proestrus. The increment in RQ of mRNAs was the highest for *Kiss1* (58.81), followed by *Gal* (6.36) and *Nts* (3.82), whereas *Cck* showed a weaker response (1.82). The downregulation of tachykinin precursor 1 (*Tac1*) suggests a declining SP production at late proestrus. Neuropeptides encoded by these genes have previously been mapped in the rostral periventricular region of the rodent brain (Simerly and Swanson, 1987; Smith and Wise, 2001; Clarkson and Herbison, 2006; Dungan et al., 2006; Ducret et al., 2010; Porteous et al., 2011; Overgaard et al., 2013; Skrapits et al., 2015). Axons arising from KP neurons of the AVPV innervate GnRH cells in mice (Smith et al., 2005; Kalló et al., 2012; Kumar et al., 2015; Yip et al., 2015) and the released neurohormone exerts an excitatory tone upon them (Han et al., 2005; Clarkson et al., 2008; Liu et al., 2011). These KP neurons also process the positive E2 feedback signal (Gottsch et al., 2009; Frazao et al., 2013; Dubois et al., 2015) and therefore, have an unequivocal role in the generation of the preovulatory GnRH-LH surge during proestrus (Dungan et al., 2006; Smith et al., 2006a; Clarkson et al., 2008). GAL is also a potent central regulator of reproduction (Merchenthaler et al., 1990; Lopez et al., 1991; Rajendren, 2002) whose expression is heavily regulated by E2 (Gabriel et al., 1993; Liposits et al., 1995). It is of note that KP neurons can co-synthesize GAL ranging from 7% seen in intact (Porteous et al., 2011) up to 87% characterizing ovariectomized, E2 replaced mice (Kalló et al., 2012). CCK synthesis also takes place in the periventricular nucleus of rodents (Simerly and Swanson, 1987; Porteous et al., 2011). In concert, CKK axons communicate with GnRH neurons in the OVLT region via CCK-1 receptors and the neuropeptide modulates their activity (Giacobini and Wray, 2007). The present finding is indicative of an increased CCK production in the MPA of the mouse brain during proestrus. A similar tendency was observed in case of *Nts* mRNA expression confirming previous reports on the estrous cycle dependent synthesis of NT (Herbison and Theodosis, 1991; Smith and Wise, 2001) in the MPA and its role in induction of the LH surge via interaction with GnRH neurons (Alexander et al., 1989a,b; Dungan Lemko et al., 2010). CCK and NT production is anatomically distinct from KP synthesis in the AVPV region without any sign of co-synthesis (Porteous et al., 2011). SP was shown to be synthesized in the AVPV region (Simerly and Swanson, 1987) and its production regulated by E2 (Okamura et al., 1994).

### Influence of Proestrus on Local GABA, Glutamate and Dopamine (DA) Neuronal Systems of the AVPV/MPOA

Similar to the rich peptidergic character of the AVPV/MPOA of the rodent brain, neurotransmitters are also synthesized herein. Glutamate (Ottem et al., 2004), GABA (Ottem et al., 2004) and DA (Simerly et al., 1997; Clarkson and Herbison, 2011) production takes place in its neurons. Neurons with dual glutamate/GABA phenotype have been identified in the area in both rats (Ottem et al., 2004) and mice (Cravo et al., 2011). These neurons also express estrogen receptors (Simerly et al., 1997; Merchenthaler et al., 2004; Zuloaga et al., 2014; Cheong et al., 2015) and via rostral projections they innervate GnRH neurons (Boehm et al., 2005). GABA- and glutamate-driven events of neurotransmission onto GnRH neurons have been the most comprehensively studied regulatory mechanisms of these neurosecretory cells (Christian and Moenter, 2010; Iremonger et al., 2010; Moenter, 2010; Penatti et al., 2010; Liu and Herbison, 2011; Taylor-Burds et al., 2015). A wide repertoire of GABA, glutamate and DA receptors characterizes the GnRH neurons (Todman et al., 2005; Vastagh et al., 2015, 2016), as a prerequisite of their control by neurotransmitters. The upregulation of solute carrier family 17, member 1 gene (*Slc17a6*) that codes for vesicular glutamate transporter 2 (VGLUT2) indicates the probability of enhanced packaging of glutamate into synaptic vesicles at the level of axon terminals in proestrus. In parallel with this, a differential regulation of the expression of various glutamate receptor mRNAs in GnRH neurons of the proestrous mice has recently been reported (Vastagh et al., 2016). Regarding the local GABA-ergic system, the expression of the GABA synthesizing enzymes (GAD1 and GAD2) did not change prior to the GnRH surge in this study. The status of the vesicular GABA pool in the AVPV/MPOA block was also monitored by the expression of the vesicular GABA transporter (*Slc32a1*) mRNA that was downregulated. The GABA catabolizing enzyme, 4-aminobutyrate aminotransferase, was slightly downregulated, too. These findings indicate that the medial preoptic GABAergic neuronal system is rather silent a few hours prior to the GnRH surge.

DA synthesis in the AVPV/MPOA has been verified long ago (Simerly and Swanson, 1987). The rate limiting enzyme of DA synthesis, TH, is co-synthesized in about 50 percent of KP neurons in the region (Clarkson and Herbison, 2011). GnRH neurons express DA receptors (Vastagh et al., 2016) and the monoamine regulates their activity (Liu and Herbison, 2013). The increased expression of *Th* mRNA in late proestrus in neurons of the AVPV/MPOA is indicative of a rising DA synthesis. In addition, the markedly increased expression of vesicular monoamine transporter 2 (*Slc18a2*) mRNA suggests that packaging of DA into synaptic vesicles within axon terminals that belong to AVPV/MPOA neurons may be accelerated. Both GABA and glutamatergic synaptic transmission mechanisms are regulated via retrograde endocannabinoid signaling utilizing cannabinoid 1 (CB1) receptors residing in the presynaptic terminals (Katona and Freund, 2012). Retrograde endocannabinoid signaling from GnRH neurons toward their GABAergic presynaptic boutons has also been substantiated earlier (Farkas et al., 2010). Therefore, it might have a special importance that CB1 receptor mRNA expression undergoes downregulation before the zenith of proestrus in the AVPV/MPOA, a main GABA and glutamate supplier of amino acid transmitter receptors of GnRH neurons.

### Altered Expression of Nuclear Hormone Receptor Genes in the Rostral Periventricular Area

Gonadal steroid hormones heavily influence the AVPV/MPOA and functions associated with this territory (Ehret and Buckenmaier, 1994; Simerly et al., 1997; Bodo et al., 2006; Kudwa et al., 2006; Nakata et al., 2016). The marked sexual dimorphism of the locus (Davis et al., 1996; Bodo et al., 2006; Kanaya et al., 2014) also exemplifies its sensitivity to gonadal steroids. The classical, estrogen receptor 1(ER-alpha; Simerly et al., 1990; Merchenthaler et al., 2004), the novel subtype, estrogen receptor 2 (ER-beta; Shughrue et al., 1997; Merchenthaler et al., 2004; Zuloaga et al., 2014) and the membrane-associated form GPER1 (Treen et al., 2016) have all been identified and mapped in different structural compartments of the region. Signaling to the region by progesterone and androgen hormones has also been demonstrated and the cellular distribution of their receptors elucidated (Handa et al., 1986; Simerly et al., 1990; Kanaya et al., 2014; Brock et al., 2015). The KP/GABA/glutamate neurons of the region are exceptional targets of gonadal hormones, including E2. During the positive E2 feedback, signaling via ERα seems to be mandatory (Smith et al., 2005; Wintermantel et al., 2006; Frazao et al., 2013; Cheong et al., 2015; Dubois et al., 2015) in KP neurons that become activated (Ducret et al., 2010; Zhang et al., 2013, 2015; Wang et al., 2016). In harmony with these events, we report here the upregulation of *Esr1* in late proestrus without any significant change in the expression of the other two estrogen receptors, *Esr2* and *Gper1*. Similarly, the expression of nuclear androgen and PR mRNAs increased. This result supports previous studies reporting the estrous cycle and gonadal hormone dependent expression of progesterone, androgen and alpha type estrogen receptors in this brain region (Handa et al., 1986; Simerly et al., 1996; Intlekofer and Petersen, 2011).

In addition, an orphan nuclear receptor called nuclear receptor related 1 (*Nr4a2*) also showed an increased expression at late proestrus. It has been shown to act as a transcriptional activator of endogenous TH (Sakurada et al., 1999) and contribute to the well-being of DA neurons in the brainstem. It might also support the activation of the DA neuron cluster of the AVPV during the positive E2 feedback.

### Modulation of Peptide and Transmitter Receptor Gene Expression in AVPV/MPOA during Proestrus

Proestrus had influence on the expression of certain peptide and neurotransmitter receptor mRNAs in the AVPV/MPOA. The upregulated mRNAs included growth hormone secretagogue receptor (*Ghsr*), μ-opioid receptor (*Oprm1*) and glutamate receptor subunit (*Gria4)* indicating that AVPV/MPOA neurons may be more forcefully modulated by ghrelin, endorphin, enkephalin and glutamate signals during proestrus. The expression of adenosine receptor 2a (*Adora2a*), neurotensin receptor 3 (*Sort1*) and arginine vasopressin receptor 1A (*Avpr1a*) mRNAs was markedly downregulated. It has been shown previously, that KP neurons in the AVPV are regulated by ghrelin (Forbes et al., 2009), opioid peptides (Zhang et al., 2013) and glutamate (Ducret et al., 2010). NT delivered onto the preoptic area was reported to influence the GnRH surge in ovariectomized estrogen-primed animals (Akema et al., 1987). Arginine vasopressin signaling via V1a receptors to the MPA of rat has been explored previously (Kalamatianos et al., 2004) finding the upregulation of the receptor expression by E2 in ovariectomized animals. In our mouse study, at late proestrus the V1a receptor mRNA undergoes downregulation.

### Effect of Proestrus on Other Regulatory Mechanisms in the AVPV/MPOA

Proestrus also resulted in altered expression of uncoupling protein 2 (mitochondrial, proton carrier, *Ucp2*). Upregulation of its mRNA implies that E2 may control mitochondria-derived reactive oxygen species generation (Paradis et al., 2003) and lipid metabolism (Dulloo and Samec, 2001) in the AVPV. The downregulation of ephrin receptor a3 (*Epha3*) in the AVPV/MPOA might reflect changes in communication between neurons and astrocytes (Murai and Pasquale, 2011). The differential expression at proestrus and the physiological role of adenosine receptor 2a (*Adora2a*) in the AVPV/MPOA await further elucidation. Aldehyde dehydrogenase 1 family, member L1 gene (*Aldh1L1*) showed a delicate down-regulation, that among others, may mirror the influence of the late proestrus specific hormonal milieu upon astrocytes (Cahoy et al., 2008).

### Predicted Interactions Among Proteins Encoded by Genes Expressed Differentially in Proestrus

The STRING v10 database and web resource was applied to demonstrate the predicted interactions of proteins encoded by the 24 genes regulated differentially in proestrus and the 48 genes analyzed in the study. This tool provided a functional interpretation of the altered gene expression via conversion of gene lists into coherent networks of genes in which edges (interactions) are scored depending on current evidences. Enrichment of higher scored edges form gene clusters. Differentially expressed genes were represented in G protein-coupled signaling, gonadal steroid signaling and synaptic vesicle cycling pathways. Although these clusters were already represented in the 48-gene network, their strengthening in the network of the differentially expressed genes may indicate the robustness and significance of changes in the synaptic transmission (signal transductions, vesicle cycling) and steroid hormone signaling in the AVPV during proestrus.

### Distribution of Neuropeptide/Transmitter System Specific Proteins in the AVPV/MPOA of the Proestrous Mice

The immunocytochemical study revealed the rostro-caudal distribution of KP, GAP, CCK and TH immunoreactive (IR) perikarya and neuronal fibers in the AVPV/MPOA of intact, late proestrous mice. The overall anatomical topography of these systems corresponds to previously published data (Simerly and Swanson, 1987; Clarkson and Herbison, 2011; Porteous et al., 2011). KP, CCK and TH immunoreactivities appeared in cell bodies of proestrous mice and the intensity of staining resembled images generated from either colchicine-treated (Porteous et al., 2011) or ovariectomized plus E2-replaced (Kalló et al., 2012) mice. In contrast, proestrus did not allow the proper visualization of galanin-synthesizing perikarya in the region despite the known estrogen-dependent expression of the neurohormone. While the labeled neurohormone producing cells occupy mainly the rostral periventricular zone, the IR neuronal processes course laterally and heavily infiltrate the entire region. The selected morphological images also exemplify that proteins, encoded by genes regulated differentially in proestrus, are expressed in the AVPV/MPOA dissected for PCR analysis.

## Conclusion

The present study provided evidence for altered expression of genes in the AVPV/MPOA of intact mice at late proestrus. We identified KP, galanin, NT, CCK, substance P as peptide, likewise, glutamate, DA and GABA as transmitter-specific phenotypes of the proestrus-regulated neurons within the region. The participation of ERα in the process strengthens further the role of the changing estrogen milieu during proestrus in the transcriptional activation of neuron assemblies of the AVPV/MPOA that have a fundamental contribution to the surge release of GnRH.

## Author Contributions

CV performed the experiments, analyzed the data and wrote the manuscript. ZL designed the experiments, supervised the project and wrote the manuscript.

## Conflict of Interest Statement

The authors declare that the research was conducted in the absence of any commercial or financial relationships that could be construed as a potential conflict of interest.

## Footnotes

^1^https://www.string-db.org
