# Supplementary material for: Impact of Proestrus on Gene Expression in the Medial Preoptic Area of Mice
Source: Front Cell Neurosci. 2017 Jul 4;11:183. doi: 10.3389/fncel.2017.00183 (PMC5495965; doi:10.3389/fncel.2017.00183)
Supplement: Supplementary file 1 [file Data_Sheet_1.docx]

Supplementary Material

Impact of proestrus on gene expression in the medial preoptic area of mice

Csaba Vastagh* and Zsolt Liposits

*** Correspondence:** Corresponding Author: vastagh.csaba@koki.mta.hu

# Supplementary Figures and Tables


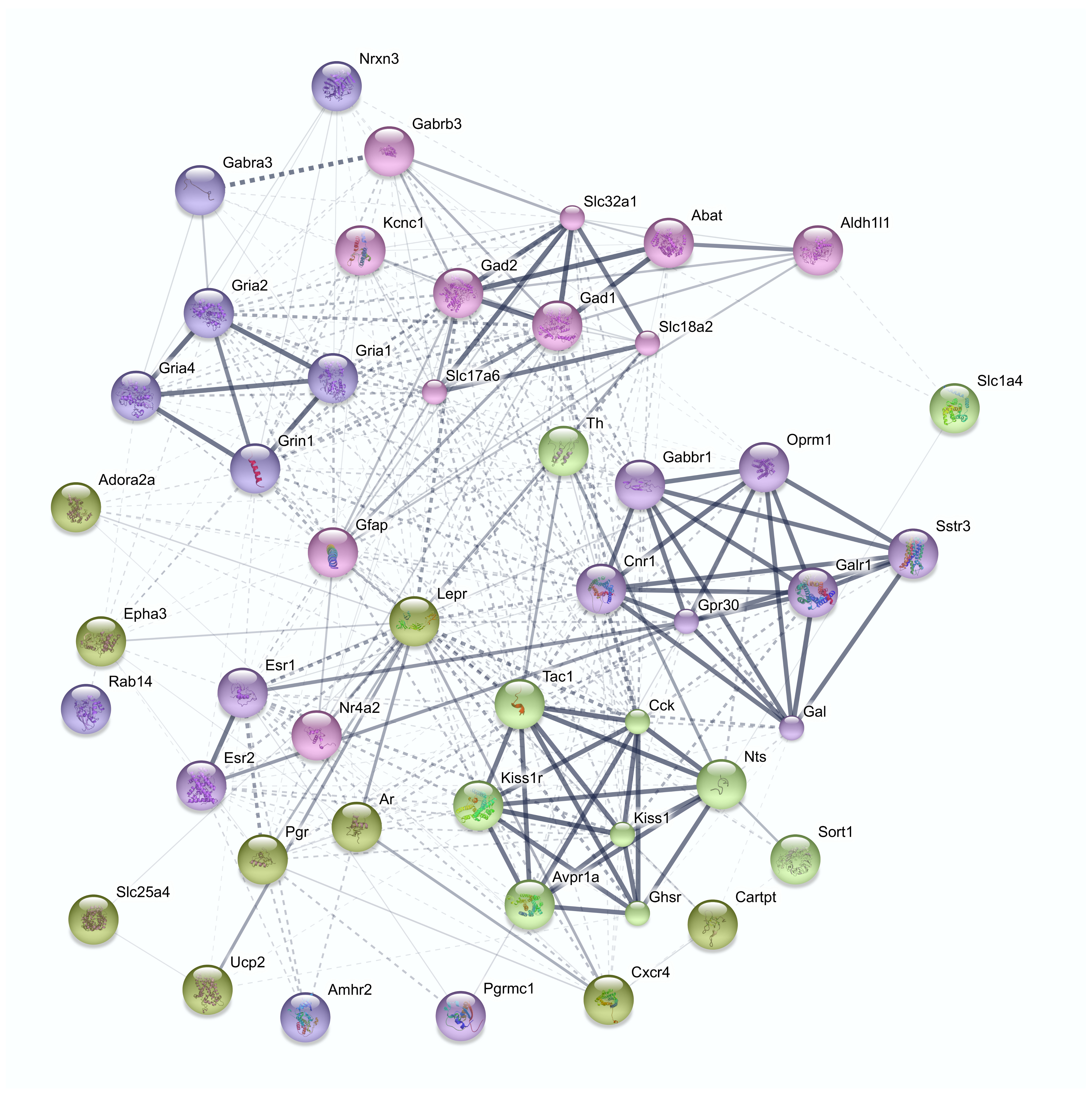


**Supplementary Figure 1.** Predicted interactions among proteins encoded by genes analyzed in the study using String 10 database and web resource (http://string-db.org). Interaction score was set to 0.15 (low confidence).
